# *CDH1*、*FANCB*和*APC*基因多态性与中国人群肺癌易感性的关系

**DOI:** 10.3779/j.issn.1009-3419.2022.102.21

**Published:** 2022-09-20

**Authors:** 联椿 苏, 华 黄, 敏 高, 永文 李, 睿峰 施, 琛 陈, 宣广 李, 光胜 朱, 红雨 刘, 军 陈

**Affiliations:** 1 832002 石河子，石河子大学医学院第一附属医院心胸外科 Department of Thoracic Surgery, First Affiliated Hospital of Medical College, Shihezi University, Shihezi 832002, China; 2 300052 天津，天津医科大学总医院肺部肿瘤外科 Department of Lung Cancer Surgery, Tianjin Medical University General Hospital, Tianjin 300052, China; 3 010030 呼和浩特，内蒙古医科大学附属医院胸外科 Department of Thoracic Surgery, the Affiliated Hospital of Inner Mongolia Medical University, Hohhot 010030, China; 4 300052 天津，天津市肺癌研究所，天津市肺癌转移与肿瘤微环境重点实验室 Tianjin Key Laboratory of Lung Cancer, Metastasis and Tumor Microenvironment, Tianjin Lung Cancer Institute, Tianjin Medical University General Hospital, Tianjin 300052, China

**Keywords:** 肺肿瘤, 单核苷酸多态性, 易感性, *CDH1*, *FANCB*, *APC*, Lung neoplasms, Single nucleotide polymorphism, Susceptibility, *CDH1*, *FANCB*, *APC*

## Abstract

**背景与目的:**

肺癌是全球癌症相关死亡的主要原因，单核苷酸多态性（single nucleotide polymorphism, SNP）是导致肺癌发生的重要因素之一，但其机制仍未阐明。本研究拟探讨*CDH1*、*FANCB*、*APC*基因SNP与肺癌遗传易感性的关系。

**方法:**

采用病例对照研究方法，收集来自天津医科大学总医院肺部肿瘤外科270例肺癌病例，同时收集445名健康志愿者的血液样本作为对照，并提取基因组DNA，使用Taqman^Ⓡ^ SNP基因分型试剂盒进行基因分型，分析*CDH1*基因rs201141645、*FANCB*基因rs754552650和*APC*基因rs149353082三个SNPs位点在中国人群中的分布。采用卡方检验和*Logistic*回归分析探索不同基因型与肺癌发病风险之间的关系。

**结果:**

*FANCB*基因rs754552650位点的AA、A/G和GG基因型的分布频率在对照组中分别为27.2%、52.6%和20.2%。AA和A/G基因型分布频率在病例组中分别为93.7%、6.3%，未检测到GG基因型。*FANCB*基因的rs754552650位点的A/G基因型在病例组和对照组中存在显著差异。携带者患肺癌的风险明显降低（OR=0.035, 95%CI: 0.020-0.062, *P* < 0.001）。*CDH1*基因rs201141645 A/C和CC基因型仅存在于对照组中。此外，在病例组中仅发现1个样本存在*APC*基因rs149353082 C/G基因型。

**结论:**

在中国人群中，*FANCB*基因rs754552650 A/G基因型携带者患肺癌的风险明显降低。

## Abstract (en)

**Background and objective:**

Lung cancer is the main cause of cancer-related death globally. Single nucleotide polymorphism (SNP) is one of the important factors leading to the occurrence of lung cancer, but its mechanism has not been elucidated. This study intends to investigate the relationship between SNPs of *CDH1*, *FANCB*, *APC* genes and lung cancer genetic susceptibility.

**Methods:**

The case-control study design was used. We collected blood samples from 270 lung cancer cases in the Department of Lung Cancer Surgery, Tianjin Medical University General Hospital, as well as blood samples from 445 healthy volunteers as controls, and extracted genomic DNA for genotyping using the Taqman^Ⓡ^ SNP genotyping kit. The distribution of three SNP loci of *CDH1* gene rs201141645, *FANCB* gene rs754552650 and *APC* gene rs149353082 in Chinese population was analyzed. Chi-square test and *Logistic* regression were used to analyze the relationship between different genotypes and the risk of lung cancer.

**Results:**

The distribution frequencies of AA, A/G and GG genotypes at rs754552650 of *FANCB* gene in the control group were 27.2%, 52.6% and 20.2%, respectively. The distribution frequencies of AA and A/G genotypes were 93.7% and 6.3% in the case group, respectively, and no GG genotype was detected. The A/G genotype of the rs754552650 locus of the *FANCB* gene was significantly different between the case group and the control group. Compared with the carriers of AA genotype, the individuals with *FANCB* rs754552650 A/G genotype had a lower risk of lung cancer (OR=0.035, 95%CI: 0.020-0.062, *P* < 0.001). *CDH1* gene rs201141645 A/C and CC genotypes only existed in the control group. In addition, only 1 sample was found to have *APC* rs149353082 genotype in the case group.

**Conclusion:**

In the Chinese population, the lung cancer risk of the individuals with *FANCB* rs754552650 A/G genotype was significantly decreased.

肺癌是导致癌症相关死亡的主要原因^[1]^，非小细胞肺癌（non-small cell lung cancer, NSCLC）是其主要类型，约占肺癌的85%。NSCLC包括腺癌（adenocarcinoma, ADC）、鳞状细胞癌（squamous cell carcinoma, SCC）、大细胞肺癌等。有关肺癌病因学的研究^[2]^已经证实，吸烟、二手烟、大麻、职业接触、非感染性呼吸道疾病史、遗传基因等与肺癌的发生有着密切的关系。其中遗传基因是我们不能忽视的一个重要原因。单核苷酸多态性（single nucleotide polymorphism, SNP）是最常见的遗传变异类型，是指由单个核苷酸的变异所引起的DNA序列的多态性。SNP可导致个体间对肺癌的易感性以及肺癌的临床病理特征出现差异。

近20年，随着高通量测序技术的不断发展，测序成本的不断下降，全基因组重测序结合全基因组关联研究（genome-wide association study, GWAS）得到了更多的发展。GWAS可以覆盖整个基因组的数百万个SNP，为我们了解人类复杂疾病的发病机制提供了更多的线索，也为SNP与肺癌之间的关联提供了更全面的认识，但仍还有许多SNP位点与肺癌的关系尚未明确，有待进一步研究。

*CDH1*（Cadherin 1）基因位于染色体16q22.1上，编码的E-钙黏蛋白（E-Cadherin）是钙依赖性细胞黏附蛋白，属于钙黏蛋白家族成员^[3]^，*CDH1*基因参与调节细胞黏附、迁移和上皮细胞增殖，其功能缺失导致细胞更容易发生侵袭与转移^[4]^，*CDH1*基因通过诱导肺癌干细胞的更新，从而促进肺癌的进展^[5]^。有研究^[6]^表明*CDH1*基因rs16260位点的C/A和C/A+AA基因型与女性肺癌中的表皮生长因子受体（epidermal growth factor receptor, *EGFR*）突变呈显著负相关，而目前关于*CDH1*基因的SNP与肺癌的研究报道较少。FANCB（FA complementation group B）蛋白是DNA修复所需的范可尼贫血（fanconi anemia, FA）核心复合物的一个组成部分^[7, 8]^，*FANCB*是唯一已知的X连锁FA基因。*FANCB*的变异约占所有男性FA病例的4%^[9]^，并且与严重的先天性异常相关。头颈部鳞状细胞癌的发展与*FANCB*的功能缺陷之间存在密切关联，*FANCB*基因产物在散发性头颈部鳞状细胞癌中显著下调^[10]^，但目前很少有研究关注*FANCB*和肺癌的关系。*APC*（APC regulator of WNT signaling pathway）是结直肠癌（colorectal cancer, CRC）中高度突变的肿瘤抑制基因，该基因的缺陷会导致家族性腺瘤性息肉病（familial adenomatous polyposis, FAP），是一种常染色体显性遗传的癌前病变，*APC*的突变和失活是结直肠肿瘤发生中几乎唯一观察到的关键和早期事件^[11]^。同时，APC蛋白是Wnt信号通路的的拮抗因子，参与细胞迁移和黏附、转录激活和细胞凋亡等过程^[12]^。当*APC*基因缺失后，可导致β-catenin磷酸化复合体合成障碍，致使细胞质内游离的β-catenin不能被泛素化降解而过量积累，引起其下游包括原癌基因*c-myc*在内的多种基因异常激活，从而导致肿瘤的发生^[13, 14]^。CRC患者中存在多种*APC*基因突变^[15, 16]^，研究^[17]^表明携带*APC*基因rs1804197位点A等位基因的受试者患CRC的风险是C等位基因携带者的2.95倍。但目前关于*APC*基因SNP位点与肺癌患者临床病理特点及易感性关系的研究较少，尚需进一步探索。

本课题组前期通过高通量测序数据筛选出三个与肺癌相关的SNP位点（*CDH1*基因rs201141645、*FANCB*基因rs754552650和*APC*基因rs149353082）。而且，目前国内外尚缺乏这三个SNPs和肺癌相关的研究。为了探究了这三个SNPs与肺癌易感性的关系，本研究收集了肺癌患者和健康志愿者的血液样本，通过Taq-man荧光定量聚合酶链式反应（polymerase chain reaction, PCR）技术对*CDH1*基因rs201141645、*FANCB*基因rs754552650和*APC*基因rs149353082在人群中进行基因分型，并研究这三个SNPs位点与肺癌遗传易感性之间的相关性。

## 1 材料与方法

### 1.1 研究人群

收集2018年11月-2021年7月在天津医科大学总医院肺部肿瘤外科通过手术或穿刺活检诊断为肺癌的270例患者的血液样本以及来自健康体检中心的445名健康志愿者的血液样本。排除标准：①有其他恶性肿瘤病史的患者；②曾接受过肺癌放疗、化疗、靶向治疗和免疫治疗的患者。天津医科大学总医院伦理委员会批准了本研究，所有受试者均确保书面知情同意参与研究。

### 1.2 DNA提取及稀释

于清晨抽取入院及健康体检中心受试者空腹静脉血4 mL置于乙二胺四乙酸（ethylenediaminetetraacetic acid, EDTA）真空抗凝管中，置于4 ℃冰箱内保存备用，并于当天采取磁珠法血液基因组DNA提取试剂盒（DP318-03，北京天根生化科技有限公司），严格按照试剂盒说明书操作提取DNA，用Nanodrop 2000紫外分光光度计测量基因组DNA的浓度和纯度，所有样本OD值（260/280）均在1.8-2.0之间，使用DNAase free水将样本稀释至15 ng/μL，然后分装于1.5 mL无酶EP管中，保存于-80 ℃冰箱内备用。

### 1.3 SNP检测

本实验应用基于TaqMan qPCR检测试剂盒（Applied Biosystems, Foster City, USA）的SNP基因分型技术以及7900 Real-Time PCR系统（Applied Biosystems, Foster City, USA）对上述三个SNPs进行基因分型实验。本次实验采用5 μL的PCR反应体系，包括2.5 μL的Taqman Master Mix、0.25 μL的引物与探针混合物以及2.25 μL的DNA样本。PCR实验所需的Taqman探针、引物及Taqman Master Mix均由美国应用生物系统公司（ABI）设计合成。在ABI 7900HT荧光定量PCR仪上进行相关PCR反应及荧光信号的读取。PCR反应的扩增条件设定为：第一步：50 ℃ 2 min，第二步：95 ℃ 10 min，第三步：95 ℃ 15 s，60 ℃ 1 min，40个循环。以SDS软件的Allelic Discrimination程序及TaqMan Genotyper软件来进行基因型分析，通过检测同一基因的不同等位基因所标记的VIC和FAM的荧光强度，来判断待测样本的基因型（红色代表无突变，绿色代表杂合突变，蓝色代表纯合突变，黑色代表未识别，未识别数据进行重复）。本研究随机抽取10%的样本进行实验，从而验证实验结果的可重复性。

### 1.4 统计学分析

数据分析基于SPSS 21.0软件。组间均数比较采用卡方检验，*P* < 0.05为差异有统计学意义。通过二元*Logistic*回归模型分析rs201141645、rs754552650和rs149353082位点SNP与肺癌遗传易感性的关联，以比值比（odds ratio, OR）及95%置信区间（confidence interval, CI）来表示关联强度。

## 2 结果

### 2.1 研究队列临床特征

研究共纳入715例，其中肺癌患者共270例，健康志愿者445名。其基本临床特征见表 1。病例组中男性144例（53.3%），女性126例（46.7%）。ADC 200例，SCC 46例，小细胞肺癌（small cell lung cancer, SCLC）15例，腺鳞癌（adenosquamous carcinoma, ASC）3例，其他类型6例。对照组中男性202例（45.4%），女性243例（54.6%）。年龄（对照组：27岁-85岁；病例组：20岁-83岁）和性别分布在对照组和病例组之间具有统计学差异（*P* < 0.05）。

**表 1:** 病例组与对照组间临床资料比较 Comparison of clinical data between case group and control group

Variable	Control group（*n*=445）	Case group（*n*=270）	*P*
Age (yr)			< 0.001
≤60	338 (76.0%)	102 (37.8%)	
> 60	107 (24.0%)	168 (62.2%)	
Gender			0.039
Female	243 (54.6%)	126 (46.7%)	
Male	202 (45.4%)	144 (53.3%)	
Histological type			
ADC		200 (74.1%)	
SCC		46 (17.0%)	
ASC		3 (1.1%)	
SCLC		15 (5.6%)	
Others		6 (2.2%)	
ADC: adenocarcinoma; SCC: squamous cell carcinoma; ASC: adenosquamous carcinoma; SCLC: small cell lung cancer. Data were calculated by *Logistic* regression with adjustment for age, gender and pathological type, where appropriate.			

### 2.2 SNP和肺癌易感性的相关性

*CDH1*基因rs201141645、*FANCB*基因rs754552650和*APC*基因rs149353082的基因分型在对照组和病例组中的等位基因频率见表 2。*CDH1*基因rs201141645位点的AA、A/C和CC基因型的分布频率在对照组中分别为72.6%、11.2%和16.2%。然而，我们在病例组中没有检测到A/C和CC基因型。*FANCB*基因rs754552650位点的AA、A/G和GG基因型的分布频率在对照组中分别为27.2%、52.6%和20.2%，AA和A/G基因型分布频率在病例组中分别为93.7%、6.3%，未检测到GG基因型。*APC*基因rs149353082位点的CC和C/G基因型在病例组中的分布频率分别为99.6%和0.4%，在对照组中我们没有检测到该位点。通过*Logistic*回归校正年龄、性别、病理类型后发现，*FANCB*基因rs754552650位点的等位基因在对照组和病例组间具有显著的差异，相比于AA基因型，A/G基因型携带者患肺癌的风险明显降低（OR=0.035, 95%CI: 0.020-0.062, *P* < 0.001），其他基因分型分布和等位基因频率在对照组和病例组间的差异均无统计学意义（*P* > 0.05）。

**表 2:** 基因多态性与肺癌发病风险相关性的*Logistic*回归分析 *Logistic* regression analysis of associations between gene polymorphisms and the risk of lung cancer

SNP	Control group (*n*=445)	Case group (*n*=270)	OR (95%CI)	*P*	OR (95%CI)^*^	*P* ^*^
*CDH1* (rs201141645)						
AA	323 (72.6%)	270 (100%)	1			
A/C	50 (11.2%)	0 (0%)		0.997		
CC	72 (16.2%)	0 (0%)		0.996		
*FANCB* (rs754552650)						
AA	121 (27.2%)	253 (93.7%)	1		1	
A/G	234 (52.6%)	17 (6.3%)	0.034 (0.020-0.057）	< 0.001	0.035 (0.020-0.062)	< 0.001
GG	90 (20.2%)	0 (0%)		0.996		0.996
*APC* (rs149353082)						
CC	445 (100%)	269 (99.6%)	1			
C/G	0 (0%)	1 (0.4%)		0.999		
Data were calculated by *Logistic* regression with adjustment for age, gender, and pathological type. ^*^adjusted by age/gender/pathological type; SNP: single nucleotide polymorphism; OR: risk factor; CI: confidence interval.						

### 2.3 临床特征分层分析

我们进一步根据临床特征年龄、性别和病理类型对*FANCB*基因rs754552650位点进行分层分析，见表 3。*FANCB*基因rs754552650的A/G基因型在≤60岁组（OR=0.045, 95%CI: 0.021-0.097, *P* < 0.001）和 > 60岁组（OR=0.027, 95%CI: 0.012-0.061, *P* < 0.001）均与肺癌易感性呈负相关。在性别分层分析中，*FANCB*基因rs754552650的A/G基因型在男性组（OR=0.008, 95%CI: 0.002-0.033, *P* < 0.001）和女性组中均与肺癌风险降低显著相关（OR=0.064, 95%CI: 0.035-0.120, *P* < 0.001）。有趣的是，根据肺癌的病理类型分层中，在病例组中，我们检测到所有的A/G基因型都来自肺腺癌患者。

**表 3:** *FANCB*基因rs754552650与肺癌风险相关性的分层分析 Stratified analyses of *FANCB* (rs754552650) for association with lung cancer risk

Variable	Control group (*n*=445)	Case group (*n*=270)	OR (95%CI)	*P*
Age				
≤60 yr				
AA	93 (20.9%)	94 (34.8%)	1	
A/G	176 (39.6%)	8 (3.0%)	0.045 (0.021-0.097)	< 0.001
GG	69 (15.5%)	0 (0%)		
> 60 yr				
AA	28 (6.3%)	159 (58.9%)	1	
A/G	58 (13.0%)	9 (3.3%)	0.027 (0.012-0.061)	< 0.001
GG	21 (4.7%)	0 (0%)		
Gender				
Female				
AA	62 (13.9%)	111 (41.1%)	1	
A/G	130 (29.2%)	15 (5.6%)	0.064 (0.035-0.120)	< 0.001
GG	51 (11.4%)	0 (0%)		
Male				
AA	59 (13.3%)	142 (52.6%)	1	
A/G	104 (23.4%)	2 (0.7%)	0.008 (0.002-0.033)	< 0.001
GG	39 (8.8%)	0 (0%)		
Histological type				
ADC				
AA	121 (27.2%)	183 (67.8%)	1	
A/G	324 (72.8%)	17 (6.3%)	0.249 (0.145-0.426)	< 0.001
SCC				
AA	121 (27.2%)	46 (17.0%)		
A/G	324 (72.8%)	0 (0%)		
ASC				
AA	121 (27.2%)	3 (1.1%)		
A/G	324 (72.8%)	0 (0%)		
SCLC				
AA	121 (27.2%)	15 (5.6%)		
A/G	324 (72.8%)	0 (0%)		
Others				
AA	121 (27.2%)	6 (2.2%)		
A/G	324 (72.8%)	0 (0%)		
In the pathological type stratified analysis, the pathological type of the case group was compared with the control group as a whole.				

## 3 讨论

肺癌是导致癌症死亡的最常见原因^[1]^。目前研究证明基因的多态性与肺癌的遗传易感性相关。因此，阐明SNP与肺癌之间的生物学机制具有重要意义。在这项研究中，我们在中国人群中收集了270例肺癌患者和445名健康志愿者，采用病例对照研究方法以探索*CDH1*基因rs201141645、*FANCB*基因rs754552650和*APC*基因rs149353082的基因分型与肺癌易感性之间的潜在关联。我们确定了*FANCB*基因rs754552650位点的A/G基因型可能作为肺癌的保护因素。迄今为止，本研究为首次评估*CDH1* rs201141645、*FANCB* rs754552650、*APC* rs149353082基因多态性与肺癌易感性之间关系的流行病学研究。

*FANCB*是一种x连锁基因，通常与FA相关。FANCB是FA核心复合物的一个组成部分，对FANCL稳定性和FANCD2泛素化至关重要，FANCD2泛素化是FA修复途径中的关键激活步骤^[7, 8]^。既往研究^[18]^表明，小鼠中*FANCB*基因的失活会减少星状细胞池并损害星状细胞的功能。然而，*FANCB*与肿瘤的相关报道较少。本研究发现*FANCB*基因rs754552650位点与肺癌的易感性呈负相关，在未来可能成为肺癌的新标志物。目前，rs754552650位点突变对*FANCB*表达及功能的影响仍不清楚，需要更进一步的研究和更大的样本来确定它们对肿瘤发展的影响。

CDH1编码E-钙黏蛋白，这是一种细胞间黏附糖蛋白，是肿瘤侵袭转移的重要抑制因子^[19, 20]^。已经有研究^[21-24]^证明，E-钙黏蛋白通过协调多个信号通路来调节基本的细胞过程，例如增殖、迁移、凋亡和侵袭。既往研究^[25]^表明*CDH1*基因rs26160的CA基因型和rs17690554的CG基因型与野生型相比是弥漫性胃癌的危险因素。*CDH1*启动子中的SNP 160 C/A是膀胱癌的危险因素^[26]^。本研究中，我们仅在对照组中发现了*CDH1*基因rs201141645位点的A/C和CC基因型，这表明它们可能是肺癌的保护因素，这需要更多的研究去探索*CDH1*和肺癌的关系。

*APC*基因位于染色体5q21-q22上，由跨越21个外显子的8, 535个核苷酸组成^[27]^。既往研究^[28]^表明，*APC*是结直肠肿瘤中的抑癌基因。*APC*基因的种系突变是导致CRC进展的主要遗传易感性事件^[29]^，同时APC也是经典Wnt信号通路的关键负调节剂，可调节胃肠道细胞的增殖和分化。有研究^[30]^表明SNP rs2019720位点GG基因型在CRC患者中比在对照组中更常见，这可能是CRC的保护因素。一项*meta*分析^[31]^表明*APC*基因多态性与CRC的风险密切相关。在我们的研究中，病例组中仅发现1个样本存在*APC*基因rs149353082 C/G基因型，这可能是由于样本量不足或与肺癌无关的罕见突变。

总之，我们的研究结果显示，在中国人群中，*FANCB*基因rs754552650基因的A/G基因型携带者患肺癌的风险明显降低，在肺癌的发生中可能作为保护因素。但本研究尚存在一定局限性：一方面，样本量相对较小，可能导致某些罕见的基因型未能检测到，因此还需要进一步扩大样本量进行研究。另一方面，由于是以医院为基础的病例对照研究，可能亦存在选择性偏差。后续工作中，除了需进一步扩大样本量进行验证外，还需从基因功能水平进一步探讨SNP参与肺癌发病的机制，同时不同基因之间、基因与环境因素之间的交互作用也有待进一步研究。
